# Mapping the Interactions of HBV cccDNA with Host Factors

**DOI:** 10.3390/ijms20174276

**Published:** 2019-09-01

**Authors:** Nur K. Mohd-Ismail, Zijie Lim, Jayantha Gunaratne, Yee-Joo Tan

**Affiliations:** 1 Department of Microbiology and Immunology, Yong Loo Lin School of Medicine, National University Health System (NUHS), National University of Singapore, Singapore 117545, Singapore; 2 Department of Medicine, Yong Loo Lin School of Medicine, National University Health System (NUHS), National University of Singapore, Singapore 119228, Singapore; 3 Institute of Molecular and Cell Biology, A*STAR (Agency for Science, Technology and Research), Singapore 138673, Singapore

**Keywords:** Hepatitis B virus, covalently closed circular DNA, host-virus interaction, drug target, screening systems

## Abstract

Hepatitis B virus (HBV) infection is a major health problem affecting about 300 million people globally. Although successful administration of a prophylactic vaccine has reduced new infections, a cure for chronic hepatitis B (CHB) is still unavailable. Current anti-HBV therapies slow down disease progression but are not curative as they cannot eliminate or permanently silence HBV covalently closed circular DNA (cccDNA). The cccDNA minichromosome persists in the nuclei of infected hepatocytes where it forms the template for all viral transcription. Interactions between host factors and cccDNA are crucial for its formation, stability, and transcriptional activity. Here, we summarize the reported interactions between HBV cccDNA and various host factors and their implications on HBV replication. While the virus hijacks certain cellular processes to complete its life cycle, there are also host factors that restrict HBV infection. Therefore, we review both positive and negative regulation of HBV cccDNA by host factors and the use of small molecule drugs or sequence-specific nucleases to target these interactions or cccDNA directly. We also discuss several reporter-based surrogate systems that mimic cccDNA biology which can be used for drug library screening of cccDNA-targeting compounds as well as identification of cccDNA-related targets.

## 1. Introduction

Viral hepatitis is a major public health concern, accounting for more than 1.3 million deaths annually [1]. Life-threatening complications such as liver cirrhosis and hepatocellular carcinoma (HCC) arise when chronic viral infections are left undiagnosed or untreated. Chronic hepatitis B virus (HBV) infection, a major contributor to viral hepatitis, is estimated to afflict nearly 300 million people globally, of which only 10% are diagnosed and an even smaller proportion are receiving treatment [2]. The availability of a prophylactic vaccine has led to a significant reduction in new HBV infections among children aged below five who are most vulnerable to developing persistent infection [1]. However, a reliable cure for individuals already living with chronic hepatitis B (CHB) is still elusive.

Currently, there are two therapeutic strategies approved for the management of chronic HBV infection, namely nucleoside/nucleotide analogs (NAs) and interferon alpha (IFN-α)/pegylated interferon (PEG-IFN) [3]. NAs suppress HBV replication and promote virological clearance by directly inhibiting viral reverse transcription. Compared to first-generation NAs lamivudine (LAM) and adefovir (ADV), newer drugs such as entecavir (ETV), tenofovir disoproximal fumarate (TDF) and tenofovir alafenamide (TAF) are more potent and have high barrier to resistance [4,5,6]. Continuous administration of these drugs can reverse cirrhosis and reduce the risk of developing end-stage liver disease and HCC, thereby improving survival in CHB patients [7,8]. NAs are generally well tolerated and ease of oral administration promotes compliance to treatment. However, the required length of treatment and the safety of these drugs in the long run are still unclear. Immune modulation and limited direct antiviral action by IFN-α/PEG-IFN over a defined treatment period resulted in higher rates of hepatitis B surface antigen (HBsAg) loss and/or seroconversion [9,10]. Following treatment with NAs for between 2 to 5 years, HBsAg loss was observed in 2–10% of hepatitis B e antigen-positive (HBeAg +ve) patients and 0–5% of HBe antigen-negative (HBeAg −ve) patients while HBsAg clearance was around 11% in both HBeAg +ve and HBeAg –ve patients 4 years after PEG-IFN treatment [11,12,13,14,15]. However, response to interferon treatment varied considerably and it is usually poorly tolerated due to adverse side effects [16,17]. Combining NA and IFN treatments either simultaneously or sequentially have shown some promise but more long-term studies are needed to determine the optimal combination for patients with different disease backgrounds or stages [18,19]. Furthermore, both these strategies are not curative as they do not primarily target HBV covalently closed circular DNA (cccDNA), which remains in infected hepatocytes and contributes to viral rebound after stopping treatment.

Targeting cccDNA is thus the next crucial step in the development of anti-HBV therapies and many reviews have comprehensively discussed different aspects of this approach [20,21,22,23,24]. Adding to that, this review will mainly focus on the regulation of HBV cccDNA activity through its interactions with host factors, as well as, recently available surrogate reporter-based systems that can be used to screen for novel cccDNA inhibitors.

## 2. HBV Covalently Closed Circular DNA

**Figure 1:**
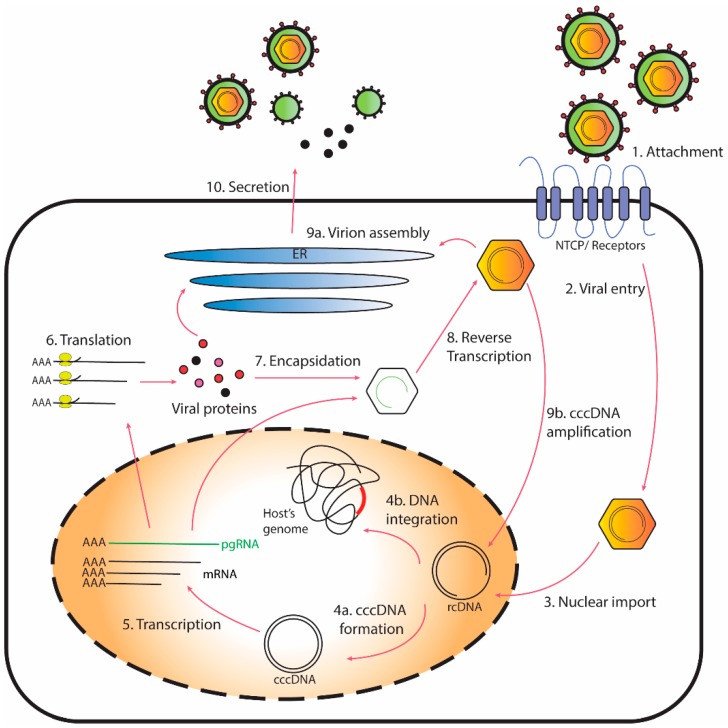
Schematic representation of the hepatitis B virus (HBV) life cycle.

The HBV life cycle includes multiple steps which are highly dependent on various host cell machinery and factors (Figure 1). Upon virus entry, the HBV genome, a partially double-stranded relaxed circular DNA (rcDNA), is transported to the nucleus of host hepatocytes. Subsequently, rcDNA is converted into cccDNA through deproteinization, removal of RNA oligomer linkage and DNA repair [25]. The cccDNA molecule serves as a template for transcription of all viral RNAs, including the pregenomic RNA (pgRNA) essential for making new copies of rcDNA to be packaged into progeny virions [26]. HBV cccDNA stably exists in the nuclei of infected cells as episomal DNA packaged into a minichromosome through interactions with cellular histone and nucleosomal proteins [27]. Although cccDNA cannot self-replicate, its levels are replenished through re-infection, de novo secondary infection and, to a lesser extent, intracellular recycling of newly synthesized rcDNA to the nucleus [28,29]. A recent study using a cell culture model of HBV infection estimates the cccDNA half-life to be around 40 days [29]. This is consistent with earlier in vivo studies that determined the half-lives of cccDNA in hepadnavirus-infected woodchucks (33 to 50 days) and ducks (35 to 57 days) [30,31]. Although the longevity of an individual cccDNA molecule has not been clearly defined, evidence suggests that cccDNA may persist for the life span of the cell [32]. In addition to its long half-life, quantification of the cccDNA level in liver biopsies of CHB patients revealed that a low copy number (median 1.5 copies per cell) is sufficient to maintain chronic infection [33]. Furthermore, reactivation of latent cccDNA may occur post-treatment leading to HBV recurrence [34,35]. Hence, cccDNA has to be eliminated from infected cells or, at least, permanently silenced in order to achieve a functional cure.

### 2.1. Blocking Formation of New cccDNA Molecules

Due to cell division and natural turnover of hepatocytes, cccDNA level in an infected liver is expected to decline over time, provided new copies are not being synthesized [36]. Formation of new cccDNA molecules through infection can be blocked at several steps including virus attachment, uptake and conversion of rcDNA into cccDNA. Identification of sodium taurocholate cotransporting polypeptide (NTCP) as a high-affinity HBV entry receptor has enabled development of and screenings for small molecule inhibitors that compete with the virus for receptor binding [37,38]. Treatment with entry inhibitor Myrcludex B, a synthetic N-myristoylated lipopeptide designed based on the HBV preS1 motif, blocked cccDNA amplification in infected human hepatocytes [39]. This drug has shown clinical efficacy against CHB and chronic hepatitis D in Phase 2 trial, although its long-term benefits and adverse effects remain to be seen [40]. Aside from its anti-HBV activity, Myrcludex B also disrupts the bile acid transporter function of NTCP as evidenced by an asymptomatic increase in bile acid levels in some patients who received the treatment [40,41]. Using a cell culture infection system, derivatives of cyclosporin A (CsA) that interact with NTCP and inhibit HBV entry without interfering with NTCP transporter function were identified [42]. This finding suggests that the HBV receptor function of NTCP may be selectively targeted to avoid potential side effects associated with inhibition of a host function.

Following virus attachment, uptake of HBV via endocytosis has been shown to be caveolin-dependent in HepaRG cells while clathrin-dependent in immortalized primary human hepatocytes (PHH) and HepG2 cells [43,44,45]. Inhibitors of clathrin-mediated endocytosis such as silibinin and chlorpromazine are capable of blocking HBV uptake [44,46]. Synthesis of new cccDNA copies via both de novo infection and intracellular recycling routes require the conversion of rcDNA into cccDNA. This step involves numerous host enzymes including DNA repair enzyme TDP2 or TDP-related proteins, flap structure-specific endonuclease 1 (FEN1), DNA polymerase alpha (Pol α) and kappa (POLK), DNA topoisomerase I (TOP1) and II (TOP2) and DNA ligases [47,48,49,50,51,52]. Chemical inhibition of these enzymes and siRNA or CRISPR/Cas9-mediated knockdown of their expression were shown to suppress cccDNA amplification. In a screen for compounds that directly target cccDNA, two disubstituted sulfonamides (DSS), CCC-0975 and CCC-0346, were identified as specific inhibitors of rcDNA conversion into cccDNA [53]. Using a similar screening strategy, hydrolyzable tannins were found to inhibit cccDNA formation as well as promote its degradation [54]. However, the mechanism and targets of inhibition by DSS and hydrolyzable tannins are still unknown. Another class of drugs that interferes with cccDNA biosynthesis is capsid assembly modulators (CAMs). These molecules target the HBV core (HBc) protein to either induce premature disassembly of nucleus-bound capsids or prevent encapsidation of newly synthesized pgRNA [55,56]. These events inhibit the generation and delivery of rcDNA to the nucleus and subsequent conversion to cccDNA.

### 2.2. Eliminating cccDNA from the Infected Liver

To eradicate HBV infection, it will not be sufficient to inhibit synthesis of new cccDNA molecules as clearance of the pre-existing cccDNA will also be required. Clearance of cccDNA can be achieved by killing infected hepatocytes or noncytolytically through the actions of pro-inflammatory cytokines and DNA-targeting enzymes. Host innate and adaptive immune responses can reduce cccDNA levels in the liver through the elimination of infected hepatocytes by natural killer cells and cytotoxic T lymphocytes [57]. Two reports suggest that certain cytokines, namely IFN-α, IFN-γ and tumor necrosis factor-α (TNF-α), and activation of lymphotoxin-β receptor (LT-βR) are capable of inducing cccDNA degradation without causing cell death through the activation of nuclear deaminases APOBEC3A and 3B [58,59]. However, due to the technical limitations of the assays employed in these studies, the claim that cytokines can induce cccDNA loss noncytopathically is slightly controversial and requires independent verification since this may have important implications for HBV cure [60]. HBV-specific immunity is usually lacking or defective in CHB patients and immune modulatory therapies are needed to mount an efficient antiviral response [61].

The use of sequence-specific nuclease technology has shown promise in inactivating or eliminating cccDNA from infected cells. Multiple studies done using zing finger nucleases (ZFNs), transcription activator-like effector nucleases (TALENs) and clustered regularly interspaced short palindromic repeats associated nuclease 9 (CRISPR/Cas9) in cell culture and animal models showed reductions in cccDNA copy number (see a recent review by Bloom et al.) [23]. Although they were designed to specifically target and disrupt HBV DNA sequences, off-target DNA cleavage events and possible increased genomic instability are still a concern [62]. Delivery of these nucleases to target cells also needs to be optimized. Nevertheless, this gene editing approach continues to be an attractive possibility for anti-HBV therapy with two CRISPR/Cas9 candidates, EBT106 and HBV, currently in preclinical testing [63].

### 2.3. Targeting cccDNA Transcriptional Activity

**Figure 2:**
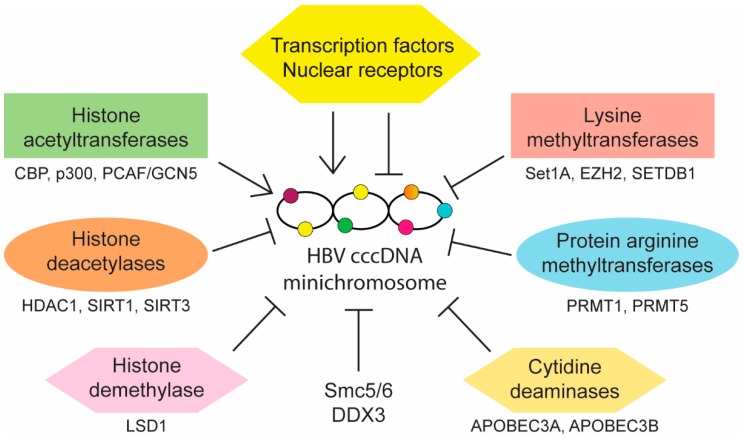
Summary of main classes of host factors that promote or inhibit covalently closed circular DNA (cccDNA) transcription.

Aside from reducing cccDNA copy number, control of HBV infection may also be achieved through the regulation of cccDNA activity. Transcription of viral pgRNA and mRNAs is regulated by four distinct promoters (preS1, preS2, core, and X) and two known enhancers (EnhI and EnhII). Multiple host transcription factors and nuclear receptors are known to bind and modulate these elements. HBV cccDNA also contains three predicted CpG islands (islands I, II, and III), of which methylation of island II has been shown to be associated with reduced cccDNA transcription in vitro and low level of circulating HBV DNA in CHB patients [64]. In addition, epigenetic modifications of cccDNA-associated histones also affect cccDNA activity. Interactions between cccDNA and host nuclear factors can either promote or inhibit its transcriptional activity (summarized in Figure 2) and understanding these interactions may provide opportunities for targeted therapy development.

#### 2.3.1. Host Factors That Positively Regulate cccDNA Activity

The cccDNA promoter and enhancer elements contain binding sites for liver-enriched as well as ubiquitous transcription factors and nuclear receptors. Those that activate viral transcription include hepatocyte nuclear factors 1, 3 and 4 (HNF1, HNF3/FoxA, HNF4), CCAAT-enhancer-binding protein (C/EBP), retinoid X receptor alpha/peroxisome proliferator-activated receptor alpha (RXRα/PPARα), Farnesoid X receptor (FXR), nuclear factor (NF1), specificity protein 1 (SP1), activator protein 1 (AP-1), TATA-binding protein (TBP), cAMP response element binding protein (CREB), octamer transcription factor 1 (Oct1), and nuclear respiratory factor 1 (NRF1) (references listed in Table 1). Involvement of transcriptional coactivators such as CREB-regulated transcriptional coactivator 1 (CRTC1) has also been demonstrated [65].

Various histone modifying enzymes including acetyltransferases (HATs), deacetylases (HDACs), lysine methyltransferases (KMTs) and protein arginine methyltransferases (PRMTs) as well as DNA methyltransferases (DNMTs) alter the methylation and acetylation status of cccDNA and its associated histones 3 and 4 (H3 and H4). Hypoacetylation of H3 and H4 correlates with low HBV replication in vitro and in vivo. Conversely, acetylation of cccDNA-bound H4 is associated with increased HBV replication and HDAC inhibitors allow maintenance of the active acetylated cccDNA minichromosome [107]. In line with this, HBx-dependent recruitment of HATs such as CREB-binding protein (CBP), p300 and p300/CBP-associated factor/general control nonderepressible 5 (PCAF/GCN5) onto cccDNA promotes histone acetylation and active transcription [92]. Besides histone acetylation, the presence of activating and repressive methylation marks on cccDNA-bound histones also determines cccDNA activity. Mapping of histone modifications in HBV-infected cells revealed high levels of activating marks such as trimethylation of lysine 4 on H3 (H3K4me3) and a corresponding absence of repressive marks such as di-or trimethylation of lysine 9 on H3 (H3K9me2 and H3K9me3) on HBV DNA, resulting in active viral transcription [108]. While a similar pattern of histone marks was observed in a subsequent study involving CHB patients, interpatient variability was evident depending on disease stage and the presence of repressive marks did not necessarily correlate with reduced viral transcription, suggesting a more complex epigenetic interplay in vivo [109]. In contrast to the earlier in vitro infection study, cccDNA was observed to be in a transcriptionally repressed state with hypoacetylated and lysine 9-methylated H3 on viral promoters 24 hours after HBV genome transfection [93]. The viral regulatory protein HBx overcomes this repression by recruiting histone demethylase lysine-specific demethylase-1 (LSD1) and KMT SET domain containing 1A (Set1A) to viral promoters. LSD1 and Set1A activate viral transcription through demethylation of repressive H3K9me2 and accumulation of activating H3K4me3, respectively [93].

#### 2.3.2. Host Restriction Factors That Directly Repress cccDNA Activity

While HBV makes use of multiple cellular proteins to complete different steps in its life cycle, it is worthwhile to note that there are also host factors that negatively regulate HBV infection, possibly as a host defense mechanism. Several transcription factors and epigenetic modifiers such as nuclear factor-kappa B (NF-κB), signal transducer and activator of transcription 1 and 2 (STAT1 and STAT2), prospero homeobox protein 1 (PROX1), enhancer of zeste homolog 2 (EZH2), Yin Yang 1 (YY1), sirtuins 1 and 3 (SIRT1 and SIRT3), PRMT1, PRMT5, HDAC1, SET domain bifurcated 1 (SETDB1), structural maintenance of chromosome 5/6 complex (Smc5/6), zinc finger and homeoboxes 2 (ZHX2), DEAD-box polypeptide 3 (DDX3) and apolipoprotein B mRNA editing catalytic polypeptide-like 3A and 3B (APOBEC3A and APOBEC3B) have been identified as host restriction factors of HBV infection (references listed in Table 1). Some of these factors directly repress the activity of cccDNA leading to reduction in viral replication and Smc5/6 is a well-characterized restriction factor (see a recent review by Livingston) [110]. Smc5/6 binds cccDNA and blocks its transcription [103]. Importantly, HBx binds to DNA-damage binding protein 1 (DDB1) which then bind to Cul4-ROC1 E3 ligase to ubiquitylate Smc5/6 for degradation so as to achieve HBV gene expression and replication [103,111]. Interestingly, a recent study found that nitazoxanide inhibits the interaction between HBx and DDB1 which restores Smc5/6 expression, leading to a reduction in viral transcription [112]. Nitazoxanide, which has been approved by the FDA for treating multiple virus and parasite infections, may be repurposed as a new therapeutic drug for HBV. As the ubiquitin-dependent degradation of Smc5/6 also requires the NEDD8 protein, pevonedistat (MLN 4924) is also able to block the degradation of Smc5/6 because it inhibits the NEDD8-activating enzyme E1 [113]. As the current HBV therapies are not able to target cccDNA, the successful use of nitazoxanide and pevonedistat to block cccDNA transcription suggests that it is feasible to develop new classes of therapies to target the activity of cccDNA.

Besides Smc5/6, ZHX2 has also been shown to restrict HBV replication [104]. Based on immunohistochemical staining of adjacent non-tumor sections from 80 HCC patients, nuclear expression of ZHX2 was found to be lower in patients who are HBV active (defined as being HBeAg positive or HBV DNA > 1000 copies/mL in serum) than those who are HBV inactive. Furthermore, overexpression of ZHX2 in Huh7-NTCP cells, which have low endogenous ZHX2 level, reduced the production of pgRNA, HBsAg and HBeAg as well as secretion of HBV core particle DNA. Conversely, knockdown of ZHX2 in HepG2-NTCP cells, which have high endogenous ZHX2 level, enhanced viral replication. It seems that ZHX2 transcriptionally represses HBV promoters as well as regulates post-translational modification (PTMs) of cccDNA-bound histones. By using cccDNA-ChIP, the authors showed that ZHX2 down-regulated PTMs associated with transcriptional activation, leading to inhibition of cccDNA transcription. Similarly, DDX3, which is a member of the DEAD-box RNA helicase family, is a host restriction factor that inhibits HBV replication at the transcriptional level [105]. The overexpression of DDX3 resulted in decrease viral RNA transcripts and the ATPase activity, but not helicase activity, of DDX3 is required for this inhibition. Interestingly, another member DDX5 did not show an inhibitory effect. By using a HBV promoter-driven reporter assay, overexpression of DDX3 inhibited the core, S1 and S2 promoter activities. Consistently, the knockdown of DDX3 in HepG2-NTCP cells increased viral replication without increasing the level of cccDNA, indicating that DDX3 represses the transcription of cccDNA.

By testing the expression of SIRT1 to 7, which are class III HDACs, SIRT3 was found to be down-regulated transcriptionally in HBV-infected HepG2-NTCP as well as HepAD38 cell line [99]. Consistently, the inhibitory effect of SIRT3 on cccDNA transcriptional activity was confirmed using ectopic SIRT3 expression as well as short hairpin RNA targeting. Although SIRT3 is localized to both mitochondrial and nucleus, it was shown that the nuclear form of SIRT3 functions as a nuclear NAD^+^-dependent HDAC and causes deacetylation of cccDNA-bound H3. Besides causing the hypoacetylation of H3K9 on cccDNA, which inhibits HBV transcription, overexpression of SIRT3 increased the recruitment of KMT SUV39H1 but decreased the recruitment of another KMT SETD1A to cccDNA in HBV-infected HepG2-NTCP cells. Consequently, the overexpression of SIRT3 caused an increase in repressive H3K9me3 and a decrease in activating H3K4me3 marks on cccDNA, resulting in an inactive chromatin structure and transcriptional silencing. Importantly, the levels of SIRT3 mRNA and SIRT3 protein on cccDNA were decreased in the liver of CHB patients when compared with inactive carriers, indicating SIRT3 acts as a HBV restriction factor in vivo. However, HBV counteracts this SIRT3-mediated repression because overexpression of HBx reduced the mRNA and protein levels of SIRT3 in Huh-7 and HepG2 cells. In HBV-infected PHH, both HBx and SIRT3 were found to bind to cccDNA and the recruitment of SIRT3 on cccDNA negatively correlated with recruitment of HBx.

PRMT5 is another host restriction factor that represses cccDNA transcription through epigenetic regulation [101]. Overexpressed PRMT5 was found to bind to cccDNA in HBV-infected dHepaRG cells and this interaction was also observed for a mutant PRMT5 lacking the methyltransferase domain. However, only wild-type but not mutant PRMT5 increased the symmetric dimethylation of cccDNA-bound H4R3, leading to decreased cccDNA transcription. Conversely, knockdown of PRMT5 decreased symmetric dimethylation of cccDNA-bound H4R3 and increased cccDNA transcription. The action of PRMT5 seems to be related to its regulation of the interaction between cccDNA and Brg-1-based human SWI/SNF chromatin remodeler as well as RNA Pol II. In a separate study, PRMT1 was identified as a binding partner of HBx [100]. While PRMT5 increased the symmetric dimethylation of cccDNA-bound H4R3, PRMT1 catalysed asymmetric dimethylation instead. Nevertheless, PRMT1 is also recruited to cccDNA in HBV infected PHH. Knockdown of PRMT1 in HepG2 cells transfected with HBV vector increased HBV transcription, indicating that PRMT1 is also a HBV restriction factor. Conversely, the overexpression of PRMT1 decreased HBV transcription and this effect is dependent on PRMT1′s methyltransferase activity because it was not observed with enzymatically-inactive PRMT1 mutant. It seems that both asymmetric and symmetric dimethylation of cccDNA-bound H4R3, which can be catalyzed by PRMT1 and PRMT5 respectively, lead to a reduction in HBV transcription. However, their relative importance in regulating cccDNA activity during HBV infection have not been determined and further studies using mouse model of infection will be required. Interestingly, HBx may counteract the repressive activity of PRMT1 because HBx is able to inhibit PRMT1′s methyltransferase activity. On the other hand, PRMT5 interacts with HBc and regulates its post-translational modifications [101,114]. However, PRMT5-HBc interaction regulates pregenomic RNA encapsidation but may not be relevant for PRMT5′s repressive activity on cccDNA.

While most of the above restriction factors regulate cccDNA epigenetically, APOBEC3A and APOBEC3B edit cccDNA and cause its degradation [58,59]. Several members of the APOBEC3 family of cytidine deaminases have previously been shown to inhibit HBV replication through deaminase-dependent and -independent mechanisms [115,116,117,118,119,120]. The exact HBV DNA species targeted by these enzymes is still unclear although a recent study suggests HBV rcDNA to be the primary substrate for deamination [121]. APOBEC3A and APOBEC3B were found to be up-regulated in differentiated HepaRG (dHepaRG) cells treated with IFN-α and LT-βR agonists respectively. Consequently, these treatments led to a reduction in the levels of cccDNA in infected dHepaRG cells as well as PHH. Both APOBEC3A and APOBEC3B colocalize with HBc in the nucleus of infected cells where they deaminate and degrade cccDNA. Furthermore, HBV core interacts with APOBEC3A and the central region of HBc (amino acids 77 to 149) is required for this interaction. In another study, APOBEC3B was also shown to interact with HBc and the presence of RNA is required for this interaction. APOBEC3B has two cytidine deaminase domains but the domain at the C-terminal is the main contributor towards inhibition of HBV replication. In addition to editing cccDNA, APOBEC3B also edits the HBV minus- and plus-strand DNAs found in the cytoplasm during reverse transcription but does not edit pgRNA. Similar to Smc5/6, an E3 ubiquitin ligase, MSL complex subunit 2 (MSL2), was found to interact with and promote ubiquitylation of APOBEC3B, resulting in its degradation and an increase in cccDNA stability [106]. Consequently, knockdown of MSL2 resulted in the up-regulation of APOBEC3B on the protein level but not at the mRNA level. In contrast, APOBEC3A expression was not affected by MSL2. Furthermore, HBx up-regulates MSL2 transcriptionally, thus counteracting the restriction of APOBEC3B on HBV replication.

HBx is a multi-functional protein known to interact with many cellular proteins and has a central role in viral replication (see a recent review by Slagle and Bouchard) [122]. As described above, HBx also regulates several host restriction factors of HBV directly or indirectly. As HBx is being expressed in infected cells, it regulates several restriction factors like Smc5/6, SIRT3, PRMT1, and APOBEC3B and counteracts their repressive actions on cccDNA, thus allowing efficient viral replication. As illustrated by the ability of nitazoxanide to inhibit viral replication by blocking the interaction between HBx and DDB1 [112], future therapies could be developed to target HBx’s ability to counteract host restriction factors of HBV.

## 3. Surrogate Systems for Screening of Anti-HBV cccDNA Drugs

Given the importance of cccDNA in HBV life cycle and its persistence in CHB patients, efficient and accurate cccDNA quantification methods are required. However, studying HBV cccDNA biology has been challenging, primarily due to the low copy number of cccDNA in infected PHH, hepatic stem cells, and NTCP-expressing hepatoma cell lines [37,123]. Currently, Southern blotting and real-time quantitative polymerase chain reaction (qPCR) are the commonly employed methods for quantifying cccDNA [124,125,126]. Detection and quantification of cccDNA by classical Southern blotting is still considered the gold standard despite its limitations. Due to high cost and laborious workflow, application of Southern blotting in high throughput screening and clinical setting is restricted. These limitations have been circumvented with the emergence of real-time qPCR approach. Compared to Southern blotting, real-time qPCR is less laborious, inexpensive, and can easily be upscaled for high throughput screening [127]. In some cases, a single copy of cccDNA can be detected using advanced PCR approaches such as Droplet Digital PCR (ddPCR) [128]. Although more sensitive, real-time qPCR is more susceptible to false positive errors due to the presence of other viral DNA intermediates, such as rcDNA and single-stranded DNA (ssDNA), which are in high abundance [129,130,131,132]. Though several strategies have been developed recently to reduce false positives, the uptake of these approaches by researchers is slow [132,133]. Given the restrictions of methods mentioned here, several surrogate systems based on recombinant cccDNA (rcccDNA) have been developed and used for screening of anti-HBV cccDNA drugs.

In an approach developed by Cai et al., an inducible reporter-based system was developed to quantify cccDNA formation through measurement of HBeAg secretion [53]. HBeAg was used as a surrogate marker for cccDNA formation in this system as the HBeAg open reading frame (ORF) and its 5′UTR were engineered to localize to opposite ends of the linearized HBV transgene cassette [53]. After formation of cccDNA, the HBeAg transcriptional cassette will be complete, allowing expression and secretion of HBeAg [53]. By quantifying the level of secreted HBeAg using ELISA, the efficiency of cccDNA formation can be determined [53]. This approach was further improved by fusing the HBeAg ORF to a human influenza hemagglutinin (HA) epitope tag [134]. This improvement enabled detection using HA tag antibody instead of the less specific HBeAg antibody that also recognizes secreted HBcAg, which is not indicative of cccDNA formation in this system [134]. Indeed, compounds that inhibit HBV cccDNA formation were identified using this system, highlighting the potential of rcccDNA technology in facilitating identification of novel cccDNA-targeting drugs [53].

In another rcccDNA-based system, the formation of rcccDNA was facilitated through Cre/loxP-mediated DNA recombination [135,136]. In this approach, the linearized cccDNA monomer was engineered to be flanked with loxP sites before introduction into HepG2 cell genome through transposon-mediated integration, forming HepG2-HBV/loxP cell lines [136]. When Cre was expressed in HepG2-HBV/loxP cells via adenoviral transduction, the rcccDNAs will be produced through Cre/loxP-mediated DNA recombination, with the loxP sites forming part of the chimeric intron. The loxP sites will be removed from the viral transcript by RNA splicing, thus leaving the function of pgRNA and viral transcripts intact [136]. In the linearized form of cccDNA, HBsAg was truncated and formation of the rcccDNA is required for HBsAg expression. Therefore, the dynamics of cccDNA can be observed through quantification of HBsAg, which now functions as a surrogate marker of cccDNA. Similarly, this system can be used to address the questions on how cccDNA is regulated and maintained in infected cells and is useful for screening cccDNA-targeting therapeutic compounds.

Despite the ability to generate cccDNA, the copy number of cccDNA formed from these approaches remained low, probably due to low recombination efficiency, thus limiting its application [135]. Using DNA minicircle technology, a new rcccDNA, termed HBVcircle, was generated. Instead of in vivo formation of cccDNA in hepatocytes, the HBVcircle DNA was formed and extracted in E. coli via integrase-mediated intramolecular recombination [137,138,139,140]. With this approach, the cccDNA generated does not contain the bacterial backbone except for a short attR sequence at the recombination site, which has no effect on viral replication [128,139]. This allows the rcccDNA to closely mimic the unmodified HBV cccDNA following transfection into cells. This is beneficial as the transcription of cccDNA from HBV plasmids containing bacterial backbone was demonstrated to be rapidly suppressed in cells [137]. In addition, when coupled with a Gaussia luciferase reporter, the expression of the rcccDNA can be conveniently monitored through luminescence, thus providing an attractive alternative for the screening of therapeutic agents against cccDNA [141].

Reporter genes such as luciferase are useful for the development of rapid, high throughput assays. However, their application in HBV studies is limited by the intolerance of insertions into the compact viral genome. To overcome this problem, Nishitsuji and colleagues produced infectious reporter HBV by co-transfecting recombinant HBV plasmid bearing the NanoLuc (NL) gene with a helper plasmid that codes for missing viral proteins [142]. Although it was first developed to monitor early steps in the HBV replication cycle such as viral entry, a subsequent study showed that when propagated with the wildtype virus, the HBV/NL reporter virus is able to mimic the entire life cycle of HBV [143]. NL activity is repressed by ETV treatment and enhanced by ectopic HBx expression, suggesting a correlation between NL activity and cccDNA level and/or transcription. However, since this system recapitulates many steps in the virus life cycle, anti-HBV agents identified using this engineered reporter HBV may not be acting directly on cccDNA and further studies would be needed to determine their mode of action.

## 4. Conclusions

The dynamic nature and complexity of cccDNA minichromosomal structure pose great challenges to the development of strategies to eliminate cccDNA and viral persistence in CHB patients. As summarized here, small molecule drugs and sequence-specific nuclease technologies have been used to target cccDNA synthesis and/or stability. In addition, multiple host factors regulating cccDNA activities have also been identified (Table 1). While some of these host factors are required for efficient cccDNA transcription, there are also many host restriction factors that directly repress the activity of cccDNA and HBV replication. More research is required to understand the interplay between HBV and different host factors. As such, several highly sensitive methods have been developed to detect and quantify cccDNA, whose level in HBV infected cells is intrinsically low. However, such assays are not suitable for high throughput screening of drugs targeting cccDNA. To overcome this, various surrogate systems that mimic cccDNA biology and have simple readouts have been developed. Some of the latest developments in this area are summarized here and these assays have been shown to be useful for drug library screening as well as identification of drug targets related to cccDNA regulation.

## Figures and Tables

**Table 1:** Host factors that promote or repress HBV cccDNA activity.

Name	Function	Effect on cccDNA	References
HNF1	Transcription factor	Activation	[66,67,68]
HNF3/FoxA	Transcription factor	Activation	[69,70,71,72,73]
HNF4	Nuclear receptor	Activation	[68,74]
C/EBP	Transcription factor	Activation	[75,76]
RXRα/PPARα	Nuclear receptor	Activation	[77,78]
FXR	Nuclear receptor	Activation	[79]
NF1	Transcription factor	Activation	[80,81]
SP1	Transcription factor	Activation	[82,83,84]
AP-1	Transcription factor	Activation	[85,86]
TBP	Transcription factor	Activation	[87,88]
CREB	Transcription factor	Activation	[89,90]
Oct1	Transcription factor	Activation	[67]
NRF1	Transcription factor	Activation	[91]
CRTC1	Transcriptional coactivator	Activation	[65]
CBP	HAT	Activation	[92]
p300	HAT	Activation	[92]
PCAF/GCN5	HAT	Activation	[92]
LSD1	Histone demethylase	Activation	[93]
Set1A	KMT	Activation	[93]
NF-κB	Transcription factor	Inhibition	[94]
PROX1	Transcription factor	Inhibition	[95]
STAT1/2	Transcription factor	Inhibition	[96,97]
EZH2	KMT	Inhibition	[97]
YY1	Transcription factor	Inhibition	[97,98]
SIRT1	Class III HDAC	Inhibition	[85,97]
SIRT3	Class III HDAC	Inhibition	[99]
PRMT1	PRMT	Inhibition	[100]
PRMT5	PRMT	Inhibition	[101]
HDAC1	Class I HDAC	Inhibition	[97]
SETDB1	KMT	Inhibition	[102]
Smc5/6	Structural maintenance of chromosomes	Inhibition	[103]
ZHX2	Transcription factor	Inhibition	[104]
DDX3	DEAD-box RNA helicase	Inhibition	[105]
APOBEC3A	Cytidine deaminase	Inhibition	[58]
APOBEC3B	Cytidine deaminase	Inhibition	[58,106]
